# Supplementary figures and images for: Longitudinal detection of somatic mutations in saliva and plasma for the surveillance of oral squamous cell carcinomas
Source: PLoS One. 2021 Sep 3;16(9):e0256979. doi: 10.1371/journal.pone.0256979 (PMC8415592; doi:10.1371/journal.pone.0256979)

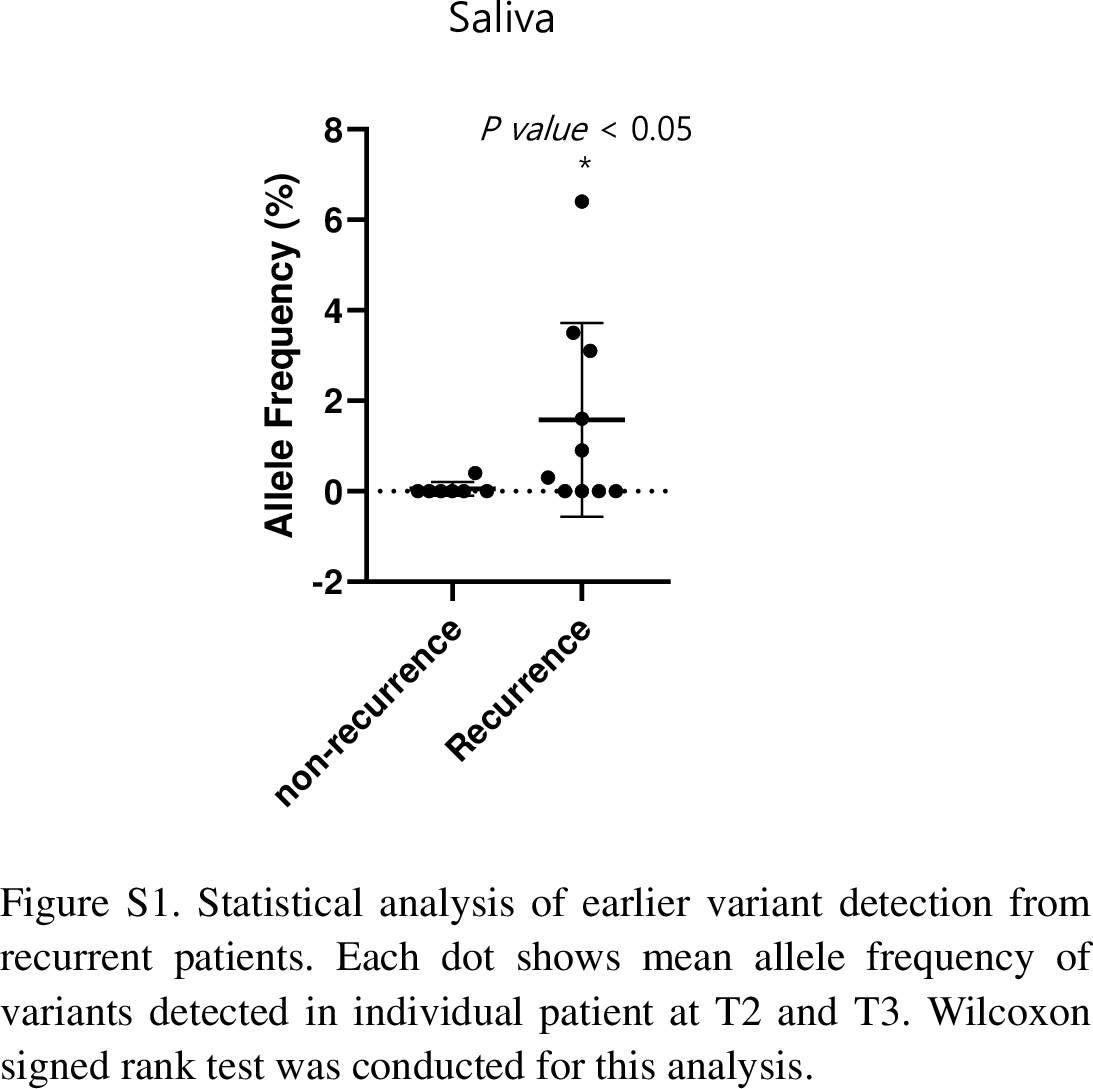

Supplement: S1 Fig — (TIF) [file pone.0256979.s001.tif]
