# Supplementary material for: Longitudinal detection of somatic mutations in saliva and plasma for the surveillance of oral squamous cell carcinomas
Source: PLoS One. 2021 Sep 3;16(9):e0256979. doi: 10.1371/journal.pone.0256979 (PMC8415592; doi:10.1371/journal.pone.0256979)
Supplement: Attachment — Submitted filename: Response to reviewers.docx [file pone.0256979.s006.docx]

Point to Point response to Reviewer’s comments

We thank the Reviewers for their critical comments and helpful suggestions, which we addressed in full to improve the impact of our data and the quality of our manuscript. As described in detail below, we have revised our manuscript in response to the Reviewers’ comments. Below is a point-to-point response to the individual comments.

Editor (Remarks to the Author)

1. Please ensure that your manuscript meets PLOS ONE's style requirements, including those for file naming. The PLOS ONE style templates can be found at https://journals.plos.org/plosone/s/file?id=wjVg/PLOSOne_formatting_sample_main_body.pdf

(Author’s Response) The format of the title page was changed according to the PLOSONE guidelines.

2. Issues about competing interest of three authors (Hae-Suk Kim, Jung Ah Park, Ju Yeong Park) and role of funder.

(Author’s Response) I would like to declare no competing interests exist, related to financial or non-financial (such as receiving funds, stocks, or shares), with Theragen Bio. Funding for conducting this study solely came from my own governmental grant written in acknowledgement (National Research Foundation of Korea (NRF) grant funded by the Korean Government). Hae-Suk Kim, Juyoung Park, and Jeonga Park, who are employees of Theragen Bio, solely contribute their scientific expertise and knowledge to conduct next-generation sequencing and bioinformatics analysis for this study. They do not have any competing interests. Also, I would like to declare that Theragen Bio did not provide any funding for this study and does not alter my adherence to PLOS ONE policies on sharing data and materials

3. We note that you are reporting an analysis of a microarray, next-generation sequencing, or deep sequencing data set. PLOS requires that authors comply with field-specific standards for preparation, recording, and deposition of data in repositories appropriate to their field. Please upload these data to a stable, public repository (such as ArrayExpress, Gene Expression Omnibus (GEO), DNA Data Bank of Japan (DDBJ), NCBI GenBank, NCBI Sequence Read Archive, or EMBL Nucleotide Sequence Database (ENA)). In your revised cover letter, please provide the relevant accession numbers that may be used to access these data. For a full list of recommended repositories

(Author’s Response) Following your recommendation, next generation sequencing data were uploaded on NCBI SRA with accession number PRJNA718901 (<https://www.ncbi.nlm.nih.gov/bioproject/PRJNA718901/>)

4. Please include details of data analysis methodology in your manuscript Methods.

(Author’s Response) We included details of data analysis methodology regarding sample collection, sequencing data analysis and data curation in Methods.

5. Please provide a sample size and power calculation in the Methods, or discuss the reasons for not performing one before study initiation.

(Author’s Response) We recorded the limitation and the reason of small sample size of the study as well as optimal sample size for repeated analysis in Discussion section. Altough Wang et al. performed liquid biopsy on 93 patients, repeated samples were only 9 patients within them with median follow up period 12 months after surgery [1].

Please consider that our study tried the earlier detection of circulating tumor DNA from 1 month to 6 months after sugery within 18 months clinical follow up.

6. In your Methods section, please provide additional information about the participant recruitment method and the demographic details of your participants. Please ensure you have provided sufficient details to replicate the analyses such as: a) the recruitment date range (month and year), b) a description of any inclusion/exclusion criteria that were applied to participant recruitment, c) a statement as to whether your sample can be considered representative of a larger population, d) a description of how participants were recruited, and e) descriptions of where participants were recruited and where the research took place.

(Author’s Response) We added detailed sample information about the recruitment range, inclusion/exclusion criteria, power and limitation of sample size (in Discussion), recruitment methods in Method section.

7. Please ensure you have discussed any potential limitations of your study in the Discussion, including study design, sample size and/or potential confounders.

(Author’s Response) We revised the manuscript in Discussion section with potential limitations of the study. As the reviewers pointed out, small sample size would be the weakest point of our study. However, longitudinal, serial liquid sample collection as well as paired solid tumor analysis were strong point of our study.

8. Please amend the manuscript submission data (via Edit Submission) to include author Eunae Sandra Cho,, Dawool Han, Jung Ah Park, Ju Yeong Park, Woong Nam, Hyung Jun Kim, In-Ho Cha.

(Author’s Response) We revised the manuscript with detailed author contribution following the guidelines of your journal. Also all authors were included during resubmission.

9. Your ethics statement should only appear in the Methods section of your manuscript. If your ethics statement is written in any section besides the Methods, please delete it from any other section.

(Author’s Response) Following the guidelines, ethics statement was written only in Methods section.

10. Please include captions for your Supporting Information files at the end of your manuscript, and update any in-text citations to match accordingly. Please see our Supporting Information guidelines for more information:

[http://journals.plos.org/plosone/s/supporting-information](about:blank).

(Author’s Response) We revised the manuscript following your journal guidelines.

Reviewer #1

1. First of all, although recurrence group includes some extensive cases (such as T4b case), I have a serious concern about the high rate of recurrence and positive margin. Please add the detailed surgical procedures (It is possible as supplemental information).

(Author's response) The limitations of this study in the discussion are the small sample size and the representativeness of the entire patient group. According to another study conducted in our institution, of 255 SCC patients, Stage I was 27.8%, Stage II 19.6%, Stage III 7.8%, Stage IVA 37.6%, Stage IVB 7.1%, in summary 47.4% for early stage and 52.6% for advanced stage [2]. In this study, the early stage was 36.4% and the advanced stage was 63.6%. LB003 contributed to a higher recurrence rate as this patient was revised to Stage IVB following reviewer's beneficial suggestion.

Summarizing the surgical procedure in relation to the positive margin, after a wide resection of the main mass during the operation, fresh-frozen biopsy was routinely performed at 5 to 7 sites in accordance with insurance criteria, and repeated until all are determined free. In the final pathologic diagnosis, the area that determines the margin cannot be completely consistent with the area where a fresh frozen biopsy was performed. In this reason, close or positive margin is reported in final pathologic diagnosis contrary to intraoperative frozen biopsy. Among the patients included in this study, patients with advanced stage had orbital, ethmoid, and pterygoid extensions where clear margin could not be obtained easily.

2. I think that LB003 is not cT2.

(Author's response) The length of the long axis measured by preoperative MRI of LB003 patient was 3.8cm, but the adjacent muscles were not considered. In fact, the medical oncologist recorded it as Stage II, and the radiation oncologist doctor recorded as Stage IVB. After careful reviewing all participants, patient LB003 was revised to Stage IVB following reviewer’s suggestion.

3. Please add the information of NM stage

(Author's response) As requested by reviewer, we have added the NM stage in Table 1.

4. LB001 is young patient. Why was adjuvant chemotherapy not performed?

(Author's response) Even if CCRT is recommended by the Oral and Maxillofacial Surgeon following NCCN guidelines, radiation alone is sometimes administered in case of the patient refuses chemotherapy by personal reason after an individual interview with medical oncologist or radiation oncologist. In this case, the patient refused platinum based chemotherapy owing to low body weight and severe anorexia.

5. How many patients with oral cancer are treated in your hospital? Please add the detailed criteria of patient enrollment. How about patient selection bias?
   (Author's response) From 2000 to 2017, the total number of SCC patients with oral cancer at the research institute was 444. Among them, 255 patients with complete medical records were considered sufficient for academic study. Recently, about 40 SCC surgeries are performed each year, and sarcoma, mucosal melanoma, and salivary gland tumors are not included in this number. We have added detailed criteria in the Methods section. Although there is an opportunity of bias due to small sample size, more patients' participation in the study was not possible due to budget overruns and time limits.

Reviewer #2:

1. Authors state in the discussion (before limitation statement) that detection of ctDNA was more efficient using plasma than saliva in recurrence cases but stated in the result that saliva provides early detection which wanes in T4 stage of sample collection where plasma values increase. This statement needs to be modified.

(Author's response) We revised the discussion section as review’s clear suggestion. As it has not yet been determined which liquid biopsy method should be applied as a standard for head and neck cancer (low-coverage whole genome sequencing, ctDNA targeted deep sequencing or HPV detection etc.), we wanted to note that the timing of sampling is clinically more important than the method.

2. In the limitation of the study, authors claim patients were in incurable state but had patients with T2 staged cancer which can be controlled or cured in many cases. The statement needs to be modified.

(Author's response) We revised that sentence to avoid confusion.

3. The limitation on the discussion should commence on a new paragraph.
   (Author's response) As reviewer’s suggestion, we revised the limitation of study in discussion section on a paragraph.

References

1.	Wang Y, Springer S, Mulvey CL, Silliman N, Schaefer J, Sausen M, et al. Detection of somatic mutations and HPV in the saliva and plasma of patients with head and neck squamous cell carcinomas. Sci Transl Med. 2015;7(293):293ra104. Epub 2015/06/26. doi: 10.1126/scitranslmed.aaa8507. PubMed PMID: 26109104; PubMed Central PMCID: PMCPMC4587492.

2.	Kim DW, Lee S, Kwon S, Nam W, Cha IH, Kim HJ. Deep learning-based survival prediction of oral cancer patients. Sci Rep. 2019;9(1):6994. Epub 2019/05/08. doi: 10.1038/s41598-019-43372-7. PubMed PMID: 31061433; PubMed Central PMCID: PMCPMC6502856.
