# Supplementary material for: Longitudinal detection of somatic mutations in saliva and plasma for the surveillance of oral squamous cell carcinomas
Source: PLoS One. 2021 Sep 3;16(9):e0256979. doi: 10.1371/journal.pone.0256979 (PMC8415592; doi:10.1371/journal.pone.0256979)
Supplement: Attachment — Submitted filename: Response to a reviewer.20210719.docx [file pone.0256979.s007.docx]

**Response to a reviewer’s comments**

Thank you for the thoughtful comments and suggestions. We revised the manuscript as following.

**1. If the authors have adequately addressed your comments raised in a previous round of review and you feel that this manuscript is now acceptable for publication, you may indicate that here to bypass the “Comments to the Author” section, enter your conflict of interest statement in the “Confidential to Editor” section, and submit your "Accept" recommendation.**

Reviewer #1: All comments have been addressed.

Reviewer #2: All comments have been addressed.

Thank you for all your confirmation.

**2. Is the manuscript technically sound, and do the data support the conclusion?**

Reviewer #1: Partly

Reviewer #2: Yes

**3. Has the statistical analysis been performed appropriately and rigorously?**

Reviewer #1: I don’t know

We agree with the reviewer. The statistical analysis is important to support for our results from the cohort. Pragmatically, we showed that ctDNA was detected approximately 4 months earlier than clinical recurrence in plasma or saliva of 3 out 6 recurred patients (Table 2 in manuscripts). Although the limitation of cohort size and sampling issue was discussed in discussion page 14, we would like to show whether variants are statistically detected in T2 and T3 which are earlier time point than clinical recurrence observation in our cohort. From this point of view, we used mean allele frequency of mutations detected in each patient at both T2 (1 month after surgery) and T3 (3 month after surgery). As you can see figure S1. below, the recurred group showed significantly positive detection of variant (mean AF, 0.05% for non-recurred versus 1.58% for recurred; median AF, 0% for non-recurred versus 0.6% for recurred; Wilcoxon signed rank test, *P* = 0.03).

Figure S1. Statistical analysis of earlier variant detection from recurrent patients. Each dot shows mean allele frequency of variants detected in individual patient at T2 and T3. Wilcoxon signed rank test was conducted for this analysis.

It is unclear whether liquid biopsy could be useful in a real practice, we deemed that the result from this small cohort can be meaningful to evaluate the clinical feasibility of liquid biopsy for oral cancer recurrence early detection. Based on current results, we will have a plan to perform a clinical validation study with our branch hospitals. Therefore, we hope that our results and the new statistical analysis using T2 and T3 data to helpful for our further study design.

Reviewer #2: Yes

Thank you for your comments.

**4. Have the authors made all data underlysing the findings in their manuscript fully available?**

Reviewer #1: Yes

Reviewer #2: Yes

**5. Is the manuscript presented in an intelligible fashion and written in standard English?**

Reviewer #1: Yes

Reviewer #2: Yes

**6. Review comments to the Author**

Reviewer #1: Why the number ctDNA detection different among patients (Figure 3c)? The authors mention that the presence of ctDNA in liquid biopsy indicated a substantial increased possibility of recurrence. Earlier ctDNA detection indicates the need for close clinical monitoring in the section of results. Please explain in more details

Response:

Thank you for your comments. We apologize that we did not clearly explained the figure. The purple triangle in Figure 3C indicates only ctDNA detection in either plasma or saliva during the study period. We attributed the missing samples to missed patient clinic visit and unmet the DNA quality to conduct the experiment in the results page 10 (Table 2, S4 table). And, the legend of figure 3C was revised.

And, we revised and added more data (Figure S1) to support the earlier ctDNA detection indicates the need for close clinical monitoring in the section of results page 11.

Reviewer #2: Yes
